# Tuberculosis in United States-Bound Follow-to-Join Asylees, 2014–2019

**DOI:** 10.4269/ajtmh.23-0233

**Published:** 2024-03-26

**Authors:** Yecai Liu, Drew L. Posey, Michelle S. Weinberg, Christina R. Phares

**Affiliations:** Division of Global Migration Health, Centers for Disease Control and Prevention, Atlanta, Georgia

## Abstract

Persons may seek asylum in the United States or at a U.S. port of entry. Principal asylees are those who are granted asylum status. Their spouse and unmarried children under 21 years of age may be granted asylum if accompanying, or following to join, the principal asylees. U.S.-bound follow-to-join asylees must undergo an overseas medical examination that includes tuberculosis (TB) screening. Culture-based overseas TB screening in U.S.-bound follow-to-join asylees has not been evaluated. We evaluated data from overseas TB screening in 19,088 arrivals of follow-to-join asylees during 2014–2019 and assessed data from their postarrival evaluation, which is recommended for those at risk for TB. Of 19,088 arrivals of follow-to-join asylees, 29 (152 cases/100,000 persons) met criteria for class B0 TB (recent completion of TB treatment overseas) and 340 (1,781 cases/100,000 persons) met criteria for class B1 pulmonary TB (chest radiograph/clinical symptoms suggestive of TB but negative sputum cultures overseas). Of 6,847 persons aged 2 to 14 years from countries with a WHO-estimated TB incidence of ≥20 cases/100,000 population/year, 408 (6.0%) were classified as class B2 latent TB infection (LTBI). Postarrival evaluations were completed in 44.8%, 51.5%, and 40.4% of persons with class B0 TB, class B1 TB, and class B2 LTBI, respectively. In conclusion, culture-based overseas TB screening in U.S.-bound follow-to-join asylees is effective in identifying those with TB (class B0 TB) or those at risk for TB (class B1 TB and class B2 LTBI). Completion of postarrival evaluation for newly arrived follow-to-join asylees was less frequent than that reported for immigrants and refugees.

## INTRODUCTION

Asylees are persons who are unable or unwilling to return to their country of nationality because of persecution or a well-founded fear of persecution due to race, religion, nationality, membership in a particular social group, or political opinion.^1^ Applicants may seek asylum in the United States or at a U.S. port of entry. Principal asylees are those who are granted asylum status in the United States. The spouse and unmarried children under the age of 21 who are listed on the principal asylee’s application for asylum but not in their grant of asylum may obtain derivative asylum status.^2^ Within 2 years of their grant of asylum, a principal asylee may petition for follow-to-join benefits for qualifying derivatives who may be located abroad or in the United States.^2^ In 2019, 46,508 persons were granted asylum, of these, 13.5% (6,270) were follow-to-join asylees located overseas and in the United States.^2^

Tuberculosis (TB) is one of the most common causes of death from infectious diseases globally.^3^ In 2022, 72.9% of new TB cases reported in the United States occurred among non-U.S.-born persons.^4^ To reduce the importation of TB into the United States, an overseas medical examination that includes TB screening is required for U.S.-bound immigrants and refugees^5^^–^^7^ and U.S.-bound follow-to-join asylees living abroad.^8^ Before 2007, a sputum smear-based algorithm was used in TB screening, but this algorithm could not identify smear-negative and culture-positive TB.^6^ In 2007, the CDC developed a new screening algorithm to include mycobacterial culture for persons with a chest radiograph suggestive of TB and directly observed therapy for those diagnosed with TB disease.^7^^,^^9^ Implementation of the culture-based screening algorithm began in 2007 and was scaled up to all countries by 2013. Culture-based overseas TB screening has been found to be effective on finding and treating U.S.-bound immigrants and refugees with TB disease,^7^ but its effectiveness in U.S.-bound follow-to-join asylees has not been assessed. We used data from CDC’s Electronic Disease Notification (EDN) database to evaluate culture-based overseas TB screening in U.S.-bound follow-to-join asylees.

## MATERIALS AND METHODS

### Overseas TB screening.

Tuberculosis screening is a major component of the mandatory medical examination for U.S.-bound immigrants and refugees^5^^–^^7^ and U.S.-bound follow-to-join asylees.^8^ The overseas medical examination is performed by “panel physicians,” licensed local physicians who are appointed by U.S. embassies and consulates.^10^ The CDC provides technical instructions and quality oversight for the examination. The culture-based algorithm, which is used in TB screening and treatment, requires all persons aged ≥15 years to have a chest radiograph and those aged 2 to 14 years in countries with a WHO-estimated TB incidence of ≥20 cases/100,000 population/year to undergo a tuberculin skin test (TST) or interferon-γ release assay (IGRA) and, if positive, to have a chest radiograph.^7^^,^^9^ Since October 2013, panel physicians have been required to make the switch from analog to digital systems for chest radiographs. Prior to 2018, TST or IGRA was used with a test for latent TB infection (LTBI). Since 2018, IGRA has been required with tests for LTBI. Persons with a chest radiograph or clinical signs or symptoms suggestive of TB must provide three sputum specimens for acid-fast bacillus microscopy and culture for mycobacteria. Use of both solid and liquid media is required for culture. Panel physicians may use molecular tests at their discretion for clinical purposes. The decision to clear an applicant with negative test results is based on the culture results. Drug susceptibility testing is required for positive cultures. Panel physicians use the liquid culture system for testing or may use molecular tests at their clinical discretion. Persons diagnosed with pulmonary TB disease are required to complete a course of directly observed therapy and have negative smear and culture results before applying for their visa to the United States.^7^

### Analysis population.

Our analytic dataset included follow-to-join asylees who were screened for TB overseas and arrived in the United States between 2014 and 2019. Data from overseas TB screening in follow-to-join asylees and postarrival evaluation in the United States for those at risk for TB were obtained from CDC’s EDN database. Culture-based overseas TB screening classifies persons as 1) class B0 TB for those diagnosed with TB disease who complete a course of directly observed therapy overseas and are cured for TB disease, 2) class B1 pulmonary TB for those who have chest radiograph, HIV, or clinical signs/symptoms suggestive of TB but negative sputum cultures overseas and no diagnosis of pulmonary TB disease, 3) class B2 LTBI for those diagnosed with LTBI overseas, and 4) no TB classification.^9^ In this analysis, we excluded persons with class B1 extrapulmonary classifications.

### Postarrival evaluation in the United States.

The CDC routinely notifies state and local health departments of arriving at-risk immigrants, refugees, and follow-to-join asylees via its EDN database^7^^,^^11^^,^^12^ and recommends that health department physicians conduct a postarrival evaluation. During the evaluation, health department physicians assign a TB diagnosis, treat TB disease, may offer preventive treatment to those with LTBI, and enter the evaluation data directly into CDC’s EDN database.^11^

### Ethics review.

This activity was reviewed by the CDC and was conducted consistent with applicable federal law and CDC policy [see, e.g., 45 C.F.R. part 46.102(l)(2), 21 C.F.R. part 56; 42 U.S.C. §241(d); 5 U.S.C. §552a; 44 U.S.C. §3501 et seq.].

### Statistical analysis.

We calculated proportions of class B0 TB, class B1 TB, and class B2 LTBI among U.S.-bound follow-to-join asylees. We applied a logistic regression model to assess risk factors for class B1 TB or class B2 LTBI in U.S.-bound follow-to-join asylees. The logistic regression model included year of arrival, sex, age, and country of birth. We did not assess risk factors for class B0 TB since convergence failed in the logistic regression (because of quasicomplete separation of data points). We calculated proportions completing the postarrival evaluation of newly arrived at-risk follow-to-join asylees and proportions of TB disease among those who completed postarrival evaluation. For those diagnosed with LTBI at postarrival evaluation, we also calculated proportions completing treatment of LTBI.

## RESULTS

### Culture-based overseas TB screening in U.S.-bound follow-to-join asylees.

Our dataset included 19,088 follow-to-join asylees who were screened for TB overseas and arrived in the United States between 2014 and 2019. Of those, 55.5% (10,588) were from the top five countries with the most follow-to-join asylees: China (29.9%), Ethiopia (9.7%), Nepal (5.4%), Cameroon (5.4%), and India (5.1%) (Table 1). Only 0.7% (141) of follow-to-join asylees were from the top three countries of birth with the most reported TB cases in the United States in 2021^13^: Mexico (0.4%), the Philippines (0.2%), and Vietnam (0.2%). Approximately half of follow-to-join asylees (52.9%) were female, and 90.0% were 2 to 44 years old (Table 1).

**Table 1:** Results of culture-based overseas TB screening in U.S.-bound follow-to-join asylees who arrived in the United States, 2014–2019

Variables	Persons Screened for TB					Persons Screened for LTBI*		
	Total (%)	Class B0 TB		Class B1 TB		Total (%)	Class B2 LTBI	
		*n*	Rate^†^	*n*	Rate^†^		*n*	%
Year of arrival								
2014	4,243 (22.2)	5	118	64	1,508	1,385	90	6.5
2015	3,405 (17.8)	8	235	56	1,645	1,128	73	6.5
2016	3,933 (20.6)	7	178	58	1,475	1,443	69	4.8
2017	2,467 (12.9)	3	122	46	1,865	942	56	5.9
2018	2,229 (11.7)	3	135	63	2,826	893	57	6.4
2019	2,811 (14.7)	3	107	53	1,885	1,056	63	6.0
Sex								
Male	8,991 (47.1)	17	189	163	1,813	3,562	223	6.3
Female	10,097 (52.9)	12	119	177	1,753	3,285	185	5.6
Age (years)								
0–1	121 (0.6)	0	0	0	0	NA	NA	NA
2–14	7,339 (38.4)	0	0	20	273	6,847	408	6.0
15–44	9,668 (50.6)	22	228	181	1,872	NA	NA	NA
45–64	1,876 (9.8)	6	320	128	6,823	NA	NA	NA
≥65	84 (0.4)	1	1190	11	13,095	NA	NA	NA
Country of birth^‡^								
China	5,706 (29.9)	19	333	103	1,805	1,866	82	4.4
Ethiopia	1,859 (9.7)	0	0	49	2,636	752	68	9.0
Nepal	1,034 (5.4)	2	193	24	2,321	393	12	3.1
Cameroon	1,025 (5.4)	1	98	15	1,463	435	15	3.4
India	964 (5.1)	1	104	23	2,386	342	8	2.3
Guatemala	787 (4.1)	1	127	2	254	389	19	4.9
Eritrea	724 (3.8)	0	0	11	1,519	288	23	8.0
Egypt	637 (3.3)	0	0	2	314	NA	NA	NA
Afghanistan	564 (3.0)	1	177	3	532	259	4	1.5
El Salvador	453 (2.4)	0	0	3	662	244	14	5.7
Other	5,335 (27.9)	4	75	105	1,968	1,879	163	8.7
TB incidence (cases/100,000 persons/year) in countries of birth								
0–19	1,300 (6.8)	0	0	8	615	NA	NA	NA
20–99	9,549 (50.0)	21	220	140	1,466	3,538	187	5.3
≥100	8,182 (42.9)	8	98	192	2,347	3,309	221	6.7
Unknown	57 (0.3)	0	0	0	0	NA	NA	NA
Total	19,088 (100)	29	152	340	1,781	6,847	408	6.0

LTBI = latent tuberculosis infection; NA = not applicable; TB = tuberculosis.

* Tests of immune response to mycobacterial antigen are required for all persons aged 2–14 years in countries with a WHO-estimated TB incidence ≥20 cases/100,000 persons/year. Such tests are not routinely required for others.

^†^ Cases per 100,000 persons.

^‡^ Top 10 countries of birth with the most follow-to-join asylees who arrived in the United States during 2014–2019.

Among the 19,088 follow-to-join asylees, 29 met the criteria for class B0 TB (152 cases/100,000 persons). Of these, none were resistant to isoniazid or rifampin, 23 (79.3%) were from the top five countries with the most follow-to-join asylees, namely, China (19, 65.5%), Ethiopia (0, 0%), Nepal (2, 6.9%), Cameroon (1, 3.4%), and India (1, 3.4%), and 22 (75.9%) and 6 (20.7%) were aged 15 to 44 and 45 to 64 years, respectively (Table 1). Persons from China had the highest rate of class B0 TB (330 cases/100,000 persons), followed by those from Nepal (193 cases/100,000 persons), Afghanistan (177 cases/100,000 persons), Guatemala (127 cases/100,000 persons), India (104 cases/100,000 persons), and Cameroon (98 cases/100,000 persons) (Table 1).

Among the 19,088 follow-to-join asylees, 340 met the criteria for class B1 TB (1,781 cases/100,000 persons); of these, 214 (62.9%) were from the top five countries with the most follow-to-join asylees, namely, China (103, 30.3%), Ethiopia (49, 14.4%), Nepal (24, 7.1%), Cameroon (15, 4.4%), and India (23, 6.8%), and 181 (53.2%) and 128 (37.6%) were aged 15 to 44 and 45 to 64 years, respectively (Table 1). Persons from Ethiopia had the highest rate of class B1 TB (2,636 cases/100,000 persons), followed by those from India (2,386 cases/100,000 persons), Nepal (2,321 cases/100,000 persons), China (1,805 cases/100,000 persons), Eritrea (1,519 cases/100,000 persons), and Cameroon (1,463 cases/100,000 persons) (Table 1). Of 340 persons with class B1 TB, all had three consecutive negative sputum cultures, 1 (0.3%) had symptoms for TB, and 15 (4.4%) were infected with HIV.

Of 6,847 follow-to-join asylees for whom LTBI screening was required (i.e., aged 2 to 14 years from countries with a WHO-estimated TB incidence of ≥20 cases/100,000 population/year), 3,712 (54.2%) did not have data for test method, 1,683 (24.6%) were tested with TST, and 1,452 (28.1%) were tested with IGRA; 408 met the criteria for class B2 LTBI (6.0%), and of these, 217 (45.3%) were from the top five countries with the most follow-to-join asylees, namely, China (82, 20.1%), Ethiopia (68, 16.7%), Nepal (12, 2.9%), Cameroon (15, 3.7%), and India (8, 2.0%) (Table 1). The rates of class B2 LTBI ranged from 1.5% among those from Afghanistan to 9.0% among those from Ethiopia (Table 1).

### Risk factors of class B1 TB in follow-to-join asylees.

The risk of class B1 TB was lower for persons aged 2 to 14 years (adjusted odds ratio [OR], 0.02; 95% CI, 0.01–0.04) and 15 to 44 years (adjusted OR, 0.13; 95% CI, 0.07–0.25) than for persons aged ≥65 years (Table 2). In comparison with persons from China, the risk of class B1 TB was higher for those from Ethiopia (adjusted OR, 2.07; 95% CI, 1.45–2.95), Nepal (adjusted OR, 1.84; 95% CI, 1.16–2.93), and India (adjusted OR, 1.64; 95% CI, 1.02–2.63) (Table 2).

**Table 2:** Evaluation of risk factors for class B1 TB and class B2 LTBI in U.S.-bound follow-to-join asylees, 2014–2019

Variables	Class B1 TB				Class B2 LTBI*			
	Persons with Class B1 TB	Persons without Class B1 TB	Crude OR (95% CI)	Adjusted OR (95% CI)	Persons with Class B2 LTBI	Persons without Class B2 LTBI	Crude OR (95% CI)	Adjusted OR (95% CI)
Year of arrival								
2014	64	4,179	0.80 (0.55–1.15)	0.81 (0.56–1.18)	90	1,295	1.10 (0.79–1.53)	1.15 (0.82–1.63)
2015	56	3,349	0.87 (0.60–1.27)	0.85 (0.57–1.25)	73	1,055	1.09 (0.77–1.55)	1.13 (0.79–1.62)
2016	58	3,875	0.78 (0.54–1.13)	0.81 (0.55–1.19)	69	1,374	0.79 (0.56–1.13)	0.83 (0.58–1.20)
2017	46	2,421	0.99 (0.66–1.13)	1.00 (0.67–1.51)	56	886	1.00 (0.69–1.44)	1.02 (0.70–1.49)
2018	63	2,166	1.51 (1.05–2.19)	1.59 (1.09–2.32)	57	836	1.08 (0.74–1.56)	1.12 (0.77–1.63)
2019	53	2,758	Reference	Reference	63	993	Reference	Reference
Sex								
Male	163	8,828	1.04 (0.84–1.28)	1.04 (0.84–1.28)	223	3,339	1.12 (0.92–1.37)	1.11 (0.90–1.36)
Female	177	9,920	Reference	Reference	185	3,100	Reference	Reference
Age (years)								
0–1	0	121	NA	NA	NA	NA	NA	NA
2–14	20	7,319	0.02 (0.01–0.04)	0.02 (0.01–0.04)				
15-44	181	9,487	0.13 (0.07–0.24)	0.13 (0.07–0.25)				
45-64	128	1,748	0.49 (0.25–0.94)	0.51 (0.26–1.01)				
>65	11	73	Reference	Reference				
Country of birth^†^								
China	103	5,603	Reference	Reference	82	1,784	Reference	Reference
Ethiopia	49	1,810	1.47 (1.04–2.08)	2.07 (1.45–2.95)	68	684	2.16 (1.55–3.02)	2.18 (1.56–3.04)
Nepal	24	1,010	1.29 (0.83–2.03)	1.84 (1.16–2.93)	12	381	0.69 (0.37–1.27)	0.68 (0.37–1.26)
Cameroon	15	1,010	0.81 (0.47–1.39)	1.27 (0.73–2.23)	15	420	0.78 (0.44–1.36)	0.79 (0.45–1.39)
India	23	941	1.33 (0.84–2.10)	1.64 (1.02–2.63)	8	334	0.52 (0.25–1.09)	0.53 (0.25–1.10)
Guatemala	2	785	0.14 (0.03–0.56)	0.26 (0.06–1.04)	19	370	1.12 (0.67–1.86)	1.13 (0.68–3.13)
Eritrea	11	713	0.84 (0.45–1.57)	1.01 (0.53–1.92)	23	265	1.89 (1.17–3.05)	1.91 (1.16–3.13)
Egypt^‡^	2	635	0.17 (0.04–0.70)	0.19 (0.05–0.77)	NA	NA	NA	NA
Afghanistan	3	561	0.29 (0.09–0.92)	0.49 (0.15–1.55)	4	255	0.34 (0.12–0.94)	0.35 (0.13–0.96)
El Salvador	3	450	0.36 (0.12–1.15)	0.65 (0.20–2.09)	14	230	1.32 (0.74–2.37)	1.36 (0.76–2.44)
Other	105	5,230	1.09 (0.83–1.44)	1.39 (1.05–1.86)	163	1,716	2.07 (1.57–2.72)	2.08 (1.58–2.74)

LTBI = latent tuberculosis infection; NA = not applicable; OR = odds ratio; TB = tuberculosis.

* Persons aged 2 to 14 years in countries with a WHO’s estimated TB incidence of ≥20 cases/100,000 persons/year.

^†^ Top 10 countries of birth with the most follow-to-join asylees who arrived in the United States during 2014–2019.

^‡^ Tests of immune response to mycobacterial antigen are not required for persons aged 2 to 14 years in Egypt.

### Risk factors of class B2 LTBI in follow-to-join asylees for whom LTBI screening was required.

In comparison with persons from China, the risk of class B2 LTBI was higher for persons from Ethiopia (adjusted OR, 2.18; 95%, CI 1.56–3.04) and Eritrea (adjusted OR, 1.91; 95% CI, 1.16–3.13) but lower for those from Afghanistan (adjusted OR, 0.35; 95% CI, 0.13–0.96) (Table 2).

### Postarrival evaluation of newly arrived at-risk follow-to-join asylees.

Postarrival evaluations were completed in 44.8% (13/29) of newly arrived follow-to-join asylees with class B0 TB, 51.5% (175/340) of those with class B1 TB, and 40.4% (165/408) of those with class B2 LTBI (Table 3). Overall, 45.4% (353) of 777 newly arrived at-risk follow-to-join asylees completed their postarrival evaluation in the United States (Table 3). At postarrival evaluation, none were diagnosed with TB disease among 13 persons with class B0 TB, 7 (4.0%) were diagnosed with TB disease (6 with culture-negative TB and 1 with unknown culture results) among 175 persons with class B1 TB, and none were diagnosed with TB disease among 165 persons with class B2 LTBI (Table 3).

**Table 3:** Results of postarrival evaluation in the United States of newly arrived at-risk follow-to-join asylees, 2014–2019

Overseas TB Classifications	Total Arrivals	Persons Who Completed Postarrival Evaluation in the United States													
		Total		TB Disease*						Inactive TB		LTBI		No TB	
		*n*	% of total arrivals	Total		Culture-Negative TB		Unknown Culture Results							
				*n*	%	*n*	%	*n*	%	*n*	%	*n*	%	*n*	%
Class B0 TB	29	13	44.8	0	0	0	0	0	0	10	76.9	0	0	3	23.1
Class B1 TB	340	175	51.5	7	4.0	6	3.4	1	0.6	36	20.6	53	30.3	79	45.1
Class B2 LTBI	408	165	40.4	0	0	0	0	0	0	1	0.6	80	48.5	84	50.9
Total	777	353	45.4	7	2.0	6	1.7	1	0.3	47	13.3	133	37.7	166	47.0

LTBI = latent tuberculosis infection; TB = tuberculosis.

* None were diagnosed with culture-positive TB at postarrival evaluation in the United States.

### Treatment of LTBI at postarrival evaluation in the United States.

Of 133 newly arrived follow-to-join asylees diagnosed with LTBI at postarrival evaluation, 122 (91.7%) were recommended for LTBI treatment, and of these, 83 (68.0%) initiated the treatment and 59 (48.4%) completed the treatment (Table 4).

**Table 4:** Results of LTBI treatment of newly arrived follow-to-join asylees who were diagnosed with LTBI at postarrival evaluation in the United States, 2014–2019

Overseas TB Classification	Persons Diagnosed with LTBI at Postarrival Evaluation	Recommendation of LTBI Treatment		Initiation of LTBI Treatment		Completion of LTBI Treatment			
		*n*	Percent of Persons Diagnosed with LTBI	*n*	Percent of Persons Recommended for LTBI Treatment	*n*	Percent of Persons Initiated LTBI Treatment	Percent of Persons Recommended for LTBI Treatment	Percent of Persons Diagnosed with LTBI
Class B1 TB	53	45	84.9	31	68.9	23	74.2	51.1	43.4
Class B2 LTBI	80	77	96.3	52	67.5	36	69.2	46.8	45.0
Total	133	122	97.1	83	68.0	59	71.1	48.4	44.4

LTBI = latent tuberculosis infection; TB = tuberculosis.

### Comparison with two previous studies of immigrants and refugees.

Table 5 compares the results of our analysis with those of a study of culture-based overseas TB screening in U.S.-bound immigrants and refugees and the results of another study of postarrival evaluation of newly arrived immigrants and refugees.^7^^,^^11^ The rates of class B0 TB (i.e., recent completion of overseas TB treatment) and class B1 TB among U.S.-bound immigrants and refugees are 258 and 3,612 cases/100,000 persons, respectively, and the rate of class B2 LTBI is 13.5% among those for whom LTBI screening was required.^7^ In comparison, our analysis found that the rates of class B0 TB and class B1 TB among follow-to-join asylees were 152 and 1,781 cases/100,000 persons, respectively, and that the rate of class B2 LTBI is 6.0% among those for whom LTBI screening was required.

**Table 5:** Comparison of overseas TB screening and postarrival evaluation of follow-to-join asylees with previous studies of immigrants and refugees

Variables	Current Analysis (*N* = 19,088)	Previous Study 1* (*N* = 1,561,460^†^)	Previous Study 2^‡^ (*N* = 90,737)
Analysis population	U.S.-bound follow-to-join asylees	U.S.-bound immigrants and refugees	Newly arrived immigrants and refugees with class B0 TB, B1 TB or B2 LTBI
Year of arrival	2014–2019	2007–2012	2013–2016
Age, years, *n* (%)			
<45	17,128 (89.7%)	1,153,364 (73.9%)	51,109 (56.3%)
≥45	1,960 (10.3%)	408,063 (26.1%)	39,628 (43.7%)
TB incidence (cases/100,000 persons/year), *n* (%)			
<100	10,849 (56.8%)	805,063 (51.6%)	Not reported
≥100	8,182 (42.9%)	735,038 (47.1%)	
Birth country, *n* (%)			
Mexico/Philippines/Vietnam	141 (0.7%)	692,978 (44.4%)	48,350 (53.3%)
India	946 (5.1%)	55,978 (3.6%)	2,502 (2.8%)
China	5,706 (29.9)	119,319 (7.6%)	4,352 (4.8%)
Other	12,295 (64.4%)	693,184 (44.4%)	35,533 (39.2%)
Rates (per 100,000 persons) of overseas TB classifications			
All persons			
Class B0 TB	152	258	NA
Class B1 TB	1,781	3,612	
Class B2 LTBI (%)	6.0%	13.5%	
Persons from India			
Class B0 TB	104	21	NA
Class B1 TB	2,386	1,410	
Class B2 LTBI (%)	2.3%	3.5%	
Persons from China			
Class B0 TB	333	111	NA
Class B1 TB	1,805	2,304	
Class B2 LTBI (%)	4.4%	11.4%	
Proportion completing a postarrival evaluation (%)	45.4%	64.0%	64.5%
Class B0 TB	44.8%	66.7% for persons with class B0 TB or B1 TB	71.1%
Class B1 TB	51.5%		66.3%
Class B2 LTBI	40.4%	58.5%	60.4%
Proportion of TB disease diagnosed in persons who completed a postarrival evaluation (%)			
Class B0 TB	0%	1.8% for persons with class B0 TB or B1 TB	0.7%
Class B1 TB	4.0%		1.6%
Class B2 LTBI	0%	0.3%	0.3%
Proportion recommending LTBI treatment among persons diagnosed with LTBI at postarrival evaluation	97.1%	Not reported	Not reported
Proportion initiating or completing treatment among persons recommended LTBI treatment (%)			
Initiation of the treatment	68.0%	Not reported	69.0%
Completion of the treatment	48.4%		40.0%

LTBI = latent tuberculosis infection; NA = not applicable; TB = tuberculosis.

* See reference 7.

^†^ Results for persons who were screened by culture-based algorithm. During the study period, class B0 TB was not created and persons who completed overseas TB treatment were reclassified as class B1 TB. Since the study reported the rate of TB disease (i.e., class B0 TB for persons who completed overseas TB treatment), we were able to recalculate the rate of class B1 TB.

^‡^ See reference 11.

In comparing country-specific results of culture-based overseas TB screening for U.S.-bound follow-to-join asylees with previously reported statistics for U.S.-bound immigrants and refugees,^7^ we focused on India and China, since only 0.8% of arrivals of follow-to-join asylees were from Mexico, the Philippines, and Vietnam. Rates of class B0 TB are 21 and 111 cases/100,000 persons among immigrants and refugees from India and China, respectively,^7^ in comparison with 104 and 333 cases/100,000 persons in our study for follow-to-join asylees from India and China, respectively. The rates of class B1 TB are 1,410 and 2,304 cases/100,000 persons among immigrants and refugees from India and China, respectively,^7^ in comparison with 2,386 and 1,805 cases/100,000 persons for follow-to-join asylees from India and China, respectively.

Our analysis found that 45.4% of follow-to-join asylees at risk for TB completed their postarrival evaluation within 1 year after their arrival in the United States. In comparison, 64.5% of newly arrived at-risk immigrants and refugees completed their postarrival evaluation.^11^ When compared with immigrants and refugees for whom LTBI treatment was recommended at postarrival evaluation,^11^ follow-to-join asylees had a similar proportion initiating the treatment (68.0% versus 69.0%) but a higher proportion completing the treatment (48.4% versus 40.0%).

## DISCUSSION

Our analysis showed that U.S.-bound follow-to-join asylees had a high risk for TB diagnosed overseas: 152 cases/100,000 persons for class B0 TB and 1,781 cases/100,000 persons for class B1 TB. We found that culture-based overseas TB screening in U.S.-bound follow-to-join asylees effectively diagnosed persons with TB disease and identified those at risk for TB. We also found that only 45.4% of newly arrived at-risk follow-to-join asylees completed their postarrival evaluation in the United States.

The epidemiology of TB in U.S.-bound follow-to-join asylees is largely unknown, since previous studies on culture-based overseas TB screening focus on U.S.-bound immigrants and refugees.^6^^,^^7^ Our analysis showed that U.S.-bound follow-to-join asylees had lower rates of class B0 TB, class B1 TB, and class B2 LTBI than U.S.-bound immigrants and refugees.^7^ These differences were likely, in part, due to various distributions of age and country of birth between U.S.-bound follow-to-join asylees and U.S.-bound immigrants and refugees. Of immigrants and refugees who are screened for TB overseas by culture-based algorithm in a previous study, 26.1% of persons are ≥45 years old and 47.1% are from countries with a WHO’s estimated TB incidence of ≥100 cases/100,000 persons/year, respectively.^7^ In our analysis of follow-to-join asylees, 10.3% of persons were ≥45 years old and 42.9% were from countries with a WHO’s estimated TB incidence of ≥100 cases/100,000 population/year. Additionally, we found that meeting criteria for class B1 TB or class B2 LTBI was associated with country of birth among follow-to-join asylees. The risk of class B1 TB was higher among follow-to-join asylees from Ethiopia, Nepal, and India than among those from China. Also in comparison with persons from China, the risk of class B2 LTBI was higher among those from Ethiopia and Eritrea but lower among those from Afghanistan. These results suggest that the yield of TB screening in U.S.-bound follow-to-join asylees depends on their country of origin, a finding that has been observed with other mobile populations.^14^

Mexico, the Philippines, Vietnam, India, and China are the top five countries of birth with the most reported TB cases in the United States.^13^ In comparison with immigrants and refugees reported in a previous study,^7^ the rates of class B0 TB were higher among follow-to-join asylees from India and China, and the rates of class B1 TB were higher among follow-to-join asylees from India but lower among those from China. These results suggest that culture-based overseas TB screening in U.S.-bound follow-to-join asylees in India and China is important and effective, since 35.0% of follow-to-join asylees were from these two countries.

Completion of postarrival evaluation for newly arrived at-risk follow-to-join asylees was less frequent than that reported for immigrants and refugees.^11^ We expected that the proportion of newly arrived at-risk follow-to-join asylees completing the postarrival evaluation would be similar to the proportion for refugees since they both have 8 months of federally funded medical assistance after their arrival in the United States and receive support from domestic refugee health programs. Missed opportunities for preventing TB in follow-to-join asylees exist since the yield and impact of overseas TB screening also depend on the number of at-risk persons who complete postarrival evaluation in the United States. Health departments and the CDC need to develop strategies to increase the proportion completing the postarrival evaluation of newly arrived at-risk follow-to-join asylees. Previous studies indicate that the proportion completing the postarrival evaluation might be improved if quarantine stations and health departments have intensive outreach.^15^^,^^16^ Completion of postarrival evaluation could also be increased if health departments have better contact information of newly arrived at-risk follow-to-join asylees.^5^ Education on TB for at-risk follow-to-join asylees by panel physicians during overseas TB screening or during the arrival process could likely increase their willingness to complete a postarrival evaluation in the United States.^16^ Other measures for improving the proportion completing the postarrival evaluation of follow-to-join asylees include health department physicians having access to a fully electronic and complete record of their overseas medical examination and treatment of TB.^11^

Latent TB infection diagnosis with discordant results in newly arrived non-U.S.-born persons is a challenge for U.S. TB control programs. A recent study has found that of 17,996 children with a positive overseas TST, 73.8% are negative when retested by IGRA in the United States; of 1,051 children with a positive overseas IGRA, 58.0% are negative when retested by IGRA in the United States.^17^ Treating LTBI in immigrants and refugees is cost-effective,^18^^,^^19^ but it is also a challenge for ensuring completion of the treatment.^20^ A study in the United States and Canada has reported that only 49.3% of foreign-born persons who initiated LTBI treatment during 2007–2008 completed the treatment.^21^ Since it has been proven to be effective,^22^^,^^23^ the CDC has recommended a shortened treatment regimen for LTBI.^24^^,^^25^ A systematic review and meta-analysis have found that shorter treatment regimens for LTBI in migrants are often associated with better outcomes.^26^ A study of treatment regimens for LTBI has reported that of patients on 9H (i.e., 9 months of daily isoniazid), 49.1% (27/55) completed treatment compared with 69.8% (187/269) of patients on 4R (4 months of daily rifampin) and 79.2% (99/125) of patients on 3HP (3 months of once-weekly isoniazid and rifapentine).^27^ But 51.6% of follow-to-join asylees for whom LTBI treatment was recommended at postarrival evaluation did not complete their treatment, suggesting that there is a need for developing new strategies. Compared with treatment outside of health departments, completion of LTBI treatment might be improved when health departments provide care and case management.^28^ Other strategies, such as offering LTBI treatment at overseas medical examinations,^29^ should be studied and considered.

Data used in our analysis have several limitations. Misclassification of TB cases might have occurred at overseas screening and postarrival evaluation in the United States. Postarrival evaluation data were unavailable for 54.6% of newly arrived at-risk follow-to-join asylees. The number of U.S.-bound follow-to-join asylees with overseas TB classifications was small for some countries of birth.

## CONCLUSION

In conclusion, culture-based overseas TB screening in U.S.-bound follow-to-join asylees is effective in identifying those with TB disease (class B0 TB) or those at risk for TB (class B1 TB and class B2 LTBI). Completion of postarrival evaluation in the United States for newly arrived follow-to-join asylees was less frequent than that reported for immigrants and refugees.
